# Analysis of Cervical Cytology Reports From Pap Smears and Their Clinical Correlation Among Women Attending a Gynecology OPD in a Tertiary Care Center in Northeast India

**DOI:** 10.7759/cureus.106635

**Published:** 2026-04-08

**Authors:** Himangshu Malakar, Bandana Bharali, Mridusmita Choudhury, Bifica Lyngdoh, Dibyajyoti Saikia, Amrita Datta, Mridul Singh

**Affiliations:** 1 Obstetrics and Gynecology, All India Institute of Medical Sciences, Guwahati, IND; 2 Pathology, All India Institute of Medical Sciences, Guwahati, IND; 3 Pharmacology, All India Institute of Medical Sciences, Guwahati, IND; 4 Oncopathology, Malabar Cancer Institute, Post Graduate Institute of Oncology Sciences and Research, Thalassery, IND

**Keywords:** cervical cancer, cervical intraepithelial neoplasia, pap smear, prevention, screening

## Abstract

Introduction: Cervical cancer remains a major global health concern among women, contributing significantly to morbidity and mortality. Timely screening, particularly through cytological methods such as the Papanicolaou (Pap) smear, plays a crucial role in the early detection of precancerous lesions and reduction of disease burden.

Aims: To evaluate the effectiveness of Pap smear cytology in detecting cervical cytological abnormalities and to identify factors associated with precancerous cervical lesions.

Methods: This study included sexually active women aged 18 years and above. Cervical samples were collected using an Ayre spatula, smeared on grease-free glass slides, and fixed in 95% ethyl alcohol for cytological evaluation.

Results: Epithelial cell abnormalities were most commonly observed in multiparous women aged 31-50 years. A substantial proportion of women were asymptomatic (188, 30.4%), while vaginal discharge was the most frequent complaint (182, 29.4%). Cytology revealed inflammatory smears in 364 (58.8%) cases, atypical squamous cells of undetermined significance (ASCUS) in 26 (4.2%), low-grade squamous intraepithelial lesion (LSIL) in 11 (1.8%), and high-grade squamous intraepithelial lesion (HSIL) in 6 (1%). Multivariate analysis showed that early age at first intercourse, contact bleeding, and cervical erosion were significantly associated with an increased risk of precancerous lesions, whereas higher educational status and a normal-appearing cervix were associated with a reduced risk. Other variables such as age, parity, socioeconomic status, oral contraceptive use, and duration of marriage showed no significant association.

Conclusions: The findings reinforce the importance of initiating cervical cancer screening from 25 to 30 years of age. Pap smear remains a simple, affordable, and non-invasive method for early detection of precancerous cervical lesions, particularly valuable in resource-limited settings like India.

## Introduction

Cervical cancer is one of the most common cause of women’s deaths worldwide. It is the second most common cancer affecting women in India, with approximately 96,922 new cases (14.7 per 100,000 women) and 60,078 deaths (9.2 per 100,000 women) reported in 2018 as a result of this malignancy [1]. According to the World Health Organization (WHO), over 80% of patients with cervical carcinoma are from low-resource settings such as India, primarily due to the lack of effective screening programs [2]. Cancer incidence rates remain one of the highest in Northeastern India. Cervical cancer accounts for up to 84.1% of genital malignancies in Assam and is the most common female malignancy in Cachar district (15.4%) [3]. The states of Mizoram, Arunachal Pradesh, Karnataka, and Nagaland were reported to have a high cervical cancer burden, with disability-adjusted life years (DALYs) exceeding 300 per 100,000 women [4].

Cervical carcinoma has a long preinvasive phase and thus can be predominantly prevented. Early detection and prompt treatment are possible with timely screening at this early stage. Invasive cervical cancer typically has a long-drawn-out natural history, over many years or even decades, from precursor lesions to invasive lesions [5]. In the form of cervical intraepithelial neoplasia (CIN), early precancerous abnormalities of the cervix can be detected years before invasive cancer occurrence, which opens a window to prevent cancer cervix [6].

Cervical cytological changes can be detected by a simple procedure called Papanicolaou (Pap) smear, which is considered as the main screening test for detecting CIN [7]. The Pap smear test is easy to carry out, cheap, and possible with fewer resources in a low setting. The total sensitivity of the Pap test in detecting a high-grade squamous intraepithelial lesion (HSIL) is 70.80% [8].

There is a need to have better cervical cancer screening and early detection plans in place, especially for Northeast India where cervical cancer is still one of the most common causes of morbidity and mortality. By exploring the correlation between cervical cytology (Pap smear results) and clinical symptoms, this study aims to provide region-specific statistics and to inform both clinical practice and public health strategies aimed at reducing the burden of cervical cancer.

## Materials and methods

This retrospective study was conducted over a period of 14 months (September 2023 to November 2024) at the Department of Obstetrics and Gynecology, All India Institute of Medical Sciences (AIIMS) Guwahati, Assam, including all women who met the inclusion and exclusion criteria (Table 1). The study was conducted after obtaining approval from the Institutional Ethics Committee of AIIMS, Guwahati, bearing reference number AIIMSG/IEC/M7/F204/2024. Clinical examination was performed on 619 patients and included a speculum examination to assess the condition of the cervix. Cervical cells were collected using an Ayre spatula and cytobrush for the Pap smear test by experienced and trained gynecologists. Data were collected using a predesigned proforma from patients’ records, including symptomatology, menstrual, obstetric, and sexual history, education, socio-economic status, occupational history, and findings from clinical examination, which included abdominal and speculum examinations. Pap smear reports were retrieved from the Department of Pathology, AIIMS, Guwahati.

**Table 1:** Inclusion and exclusion criteria of the women included in the retrospective study.

Inclusion criteria	Exclusion criteria
Medical records of women aged 18 to 65 years who attended the gynecological OPD between September 2023 and November 2024 and in whom a Pap smear was performed. Records with complete documentation of Pap smear results and clinical findings.	Pap smear performed during pregnancy

Cervical cytology results were reported according to the Bethesda System 2014. Pap smear reports were broadly categorized into negative for intraepithelial neoplasia (NILM) and epithelial cells abnormality (ECA). Women with positive Pap test results, including atypical squamous cells of undetermined significance (ASCUS), low-grade squamous intraepithelial lesion (LSIL), and high-grade squamous intraepithelial lesion (HSIL), were followed up according to the Federation of Obstetric and Gynecological Societies of India (FOGSI) Guidelines for Cervical Cancer Prevention and Management (June 2024).

## Results

A total of 619 women who visited the gynecology outpatient department between September 2023 and November 2024 were retrospectively analyzed. The forest plot demonstrates that certain clinical findings (cervical erosion, bleed on touch, white discharge, hypertrophied cervix) and sociodemographic factors (early marriage, low education, low socioeconomic status) are significantly associated with higher odds of abnormal cervical cytology. In contrast, factors such as a healthy cervix on examination, higher educational attainment, and urban residence were associated with a lower risk of developing precancerous cervical lesions.

Predictors for the study

**Figure 1:**
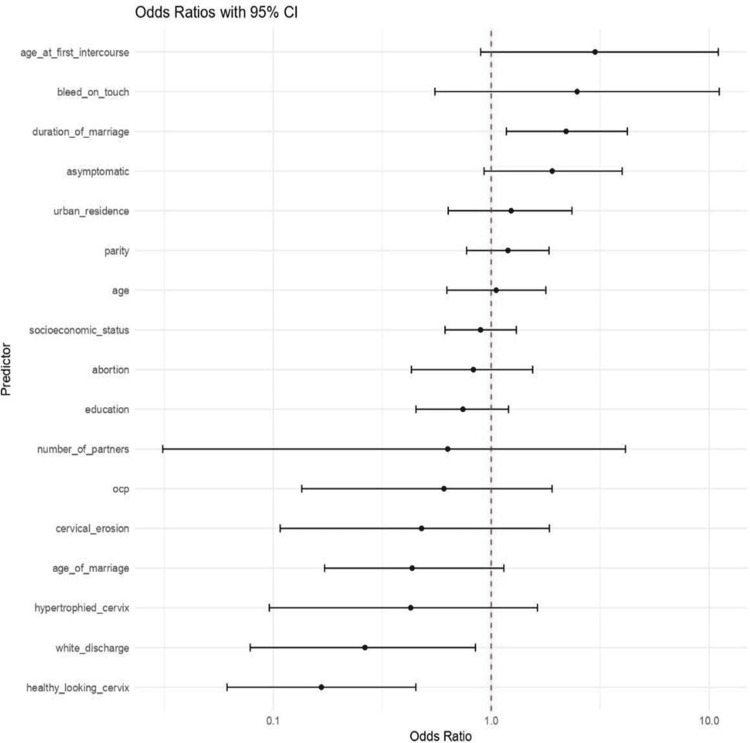
Forest plot of odd ratios showing various predictors of abnormal cervical cytology findings. Odds ratios calculated using binary logistic regression. *P* < 0.05 was considered significant.

The predictors of the study are shown in Figure 1.

Clinical findings: healthy-looking cervix, white discharge, hypertrophied cervix, cervical erosion, bleeds on touch

Demographic factors: age, parity, urban residence, socioeconomic status, education

Reproductive factors: age at marriage, age at first intercourse, duration of marriage, number of partners, contraception (OCP), abortion

Multivariate logistic regression analysis was performed to determine predictors of abnormal cervical cytology. Cervical erosion (odds ratio (OR) 2.31, 95% confidence interval (CI) 1.21-4.42, *P *= 0.01), bleed on touch (OR 3.12, 95% CI 1.02-9.51, *P *= 0.04), and early age at first intercourse (OR 1.89, 95% CI 1.05-3.41, *P* = 0.03) showed statistically significant association. Higher education was protective (OR 0.54, 95% CI 0.31-0.92, *P* = 0.02). Chi-square test and logistic regression were used.

Most women were asymptomatic (188, 30.4%). Among the presenting symptoms, vaginal discharge was the most common, observed in 182 (29.4%) of women; abdominal pain in 29 (4.7%); postcoital bleeding in 9 (1.5%); irregular menstrual cycles in 25 (4%); and postmenopausal bleeding in 24 (3.9%) of women (Table 2).

**Table 2:** Presenting symptoms of women who underwent Pap smear testing. Data are presented as *n* (%). The chi-square test was used for categorical variables, and the Student’s t-test for continuous variables. A *P*-value of <0.05 was considered statistically significant.

Symptoms	*n* (*N* = 619)	%
Asymptomatic	188	30.4
White discharge per vaginum	182	29.4
Pain in abdomen	29	4.7
Postcoital bleeding	9	1.5
Irregular cycle	25	4
Postmenopausal bleeding	24	3.9
Something coming out through per vaginum	33	5.3
Frequency of micturition	0	0
Others	115	18.58

Table 3 shows that, on per speculum examination, white discharge was observed in 148 (23.9%) participants, cervical hypertrophy in 30 (4.8%), cervical erosion in 37 (5.97%), and cervical bleeding on touch in 2 (0.32%) participants. Cases with an unhealthy cervix and cervical bleeding on touch showed epithelial abnormalities.

**Table 3:** Per speculum examination findings of patients who underwent Pap smear testing. Data are presented as *n* (%). The chi-square test was used for categorical variables, and the Student’s t-test for continuous variables. A *P*-value of <0.05 was considered statistically significant.

Findings	*n* (*N* = 619)	%
Healthy looking cervix	353	57.0
White discharge per vaginum	148	23.9
Hypertrophied cervix	30	4.8
Cervical erosion	37	5.97
Bleed on touch cervix	2	0.32
Ectropion of cervix	12	1.9
UV prolapse/cystocele	35	5.65
Others	2	0.32

Table 4 shows that atrophic smears were observed in 20 (3.2%) participants and inflammatory smears in 364 (58.8%). Epithelial abnormalities - ASCUS, LSIL, and HSIL - were found in 26 (4.2%), 11 (1.8%), and 6 (1%) of women, respectively. Unsatisfactory samples accounted for 64 (10.2%), while the remainder had adequate sample reporting.

**Table 4:** Distribution of participants according to epithelial cell abnormalities. Data are presented as *n* (%). The chi-square test was used, and a *P*-value of <0.05 was considered statistically significant. NILM, negative for intraepithelial lesion or malignancy; ASCUS, atypical squamous cells of undetermined significance; LSIL, low-grade squamous intraepithelial lesion; HSIL, high-grade squamous intraepithelial lesion; SCC, squamous cell carcinoma

Pap smear report	*n* (*N* = 619)	%
Unsatisfactory	64	10.2
NILM		
Inflammatory	364	58.8
Atrophic smear	20	3.2
No other changes	111	17.9
Epithelial cell abnormalities		
ASCUS	26	4.2
LSIL	11	1.8
HSIL	6	1
SCC	0	0
Others	17	2.74

## Discussion

Cervical cancer remains a largely preventable cause of mortality among women worldwide. The Pap smear serves as a simple, cost-effective screening tool for detecting cytological changes in the cervical transformation zone, most commonly induced by human papillomavirus (HPV) infection. Evidence suggests that screening even once after the age of 35 can reduce the risk of cervical cancer mortality by 70%, while screening every five years can reduce it by more than 85% [9]. Despite this, over 1.5 billion women globally have never been screened for cervical cancer [10]. According to the American Cancer Society (2012), the Pap smear test is a routine cancer screening method that should be done every three years, and a Pap smear with an HPV DNA test is recommended as a screening method every five years [11]. The scenario in Assam is equally concerning: ICMR (2021) reported that only 0.2% of women aged 30-49 years have ever undergone screening for cervical cancer [12]. Consequently, most patients present to gynecological outpatient departments at advanced stages of the disease.

In the present study, most cases (364, 58.8%) had inflammatory smears, which is consistent with the results reported by Mishra et al. [13]. Additional Indian reports have documented vaginal inflammation in women with limited knowledge of reproductive health and poor genital hygiene [13]. Atrophic smears were observed in 3.2% of cases, particularly among postmenopausal women, which was comparable to previous reports correlating atrophic cytology with estrogen depletion. These findings stress the significance of correlation of cytological patterns with clinical and hormonal status during interpretation [14].

Epithelial cell abnormalities were reported in 7% of women, with the following categories: ASCUS (*n* = 111, 4.2%), LSIL (*n* = 47, 1.8%), and HSIL (*n* = 25, 1%). These rates are similar to and consistent with other studies from India where abnormal cytology has been reported between 5% and 8% [15]. The high prevalence of low-grade lesions (ASCUS and LSIL) suggests that most of the abnormalities are in an early (and possibly curable) stage, which corroborates the fact that opportunistic programs can frequently detect these precancerous alterations before they become cancer. Interestingly, no cases of squamous cell carcinoma were identified, a finding likely due to early detection and adherence to established referral and practice guidelines.

The most frequent symptom was white vaginal discharge (29.4%), followed by abdominal pain (4.7%) and postcoital bleeding (1.5%). A considerable number of women (30.4%) were asymptomatic. These results are similar to other studies, which demonstrate a high incidence of asymptomatic women with abnormal cytology, re-enforcing the requirement for screening even in the absence of clinical symptoms [16].On per speculum examination, white discharge was the most common finding, followed by cervical hypertrophy and erosions - conditions that predispose to cervical inflammation and subsequent epithelial changes.

Multivariate analysis presents the ORs with 95% CIs for sociodemographic, reproductive, and clinical factors predicting precancerous cervical lesions. Of the variables examined, age at first sexual intercourse, bleeding on contact, and cervical erosion demonstrated significant associations with increased odds of precancerous cervical lesions, indicating their importance as key risk factors. These findings correspond with available data showing that an earlier onset of sexual activity lengthens the period of exposure to oncogenic HPV, whereas persistent irritation and epithelial damage (as observed in erosion or contact bleeding) may promote infection and dysplasia [17]. Conversely, a higher level of education and a healthy-appearing cervix were identified as protective factors, with ORs less than 1. Predictors such as age, parity, socioeconomic status, oral contraceptive use, and duration of marriage did not show statistically significant associations, as their CIs included the null value (OR = 1). These findings suggest that early sexual exposure and certain clinical findings increase the risk of cervical pathology, whereas education and a healthy cervix may confer protection against precancerous changes.

In contrast to some earlier reports, the present study demonstrated a protective association of oral contraceptive pill (OCP) use with abnormal cervical cytology. Women who reported OCP use showed lower odds of epithelial cell abnormalities compared to non-users.

Rather than the direct biological effects of the contraceptives, the apparent association between OCP use and reduced cytological abnormalities may be due to behavioral and sociodemographic factors. Users of oral contraceptives are more likely to undergo regular gynecologic examinations and receive counseling, as they are generally more engaged with healthcare. Thus, cervical intraepithelial changes can be detected and controlled early, which will ultimately reduce the risk of cytological abnormalities. Therefore, it is possible that the protective effect observed is a result of different health-seeking behaviors rather than the action of OCPs on cervical tissue.

More thorough and definitive insights into this association may be obtained by conducting additional research that incorporates HPV DNA testing and examines OCP exposure by duration. Combining HPV DNA testing with routine Pap smear examinations can substantially increase diagnostic precision and strengthen preventive initiatives.

The results of this study highlight the interaction of clinical, reproductive, and sociodemographic factors in cervical pathology and are in line with earlier national data [18]. A window of opportunity for preventive intervention is suggested by the predominance of inflammatory and low-grade lesions. The significance of community-based awareness programs and education in lowering the incidence of cervical cancer in Northeast India is further supported by the strong correlation between protective outcomes and educational status.

The burden of cervical cancer-related morbidity and mortality can be significantly decreased by improving region-specific screening programs and incorporating cervical cytology into standard gynecological care.

Limitations of the study 

This study has certain limitations that should be considered while interpreting the findings. First, the retrospective design relies on previously recorded medical data, which may be subject to incomplete documentation. 

Second, as a single-center study at a tertiary-care institute in Northeast India, the findings may not be fully generalizable, particularly to women in rural areas or with limited access to healthcare.

Furthermore, the study relied solely on cytological evaluation without incorporation of HPV DNA testing or histopathological confirmation.

Despite these limitations, the study provides valuable insights into cervical cytology patterns and associated risk factors, highlighting the importance of strengthening screening programs in the region.

## Conclusions

The findings reinforce the importance of initiating cervical cancer screening from 25 to 30 years of age. Although newer modalities like HPV DNA testing offer higher sensitivity for detecting high-risk HPV infections and can be used as a primary or complementary screening tool, Pap smear remains a simple, affordable, and non-invasive method for early detection of precancerous cervical lesions, particularly valuable in resource-limited settings like India.
